# The genetic architecture of psychophysiological phenotypes

**DOI:** 10.1111/psyp.12355

**Published:** 2014-11-11

**Authors:** Marcus R Munafò, Jonathan Flint

**Affiliations:** a MRC Integrative Epidemiology Unit (IEU), University of BristolBristol, UK; b UK Centre for Tobacco and Alcohol Studies and School of Experimental Psychology, University of BristolBristol, UK; c Wellcome Trust Centre for Human Genetics, University of OxfordOxford, UK

**Keywords:** Genetics, Genetic architecture, Intermediate phenotypes, Genome-wide association study

## Abstract

It is now clear that almost all complex traits have a highly polygenic component; that is, their genetic basis consists of relatively frequent risk alleles at a very large number of loci, each making a small contribution to variation, or disease susceptibility. This general conclusion appears to hold for intermediate phenotypes. Therefore, we should not expect these phenotypes to be associated with substantially larger effect sizes than conventional phenotypes. Instead, their usefulness is likely to lie in understanding the mechanism underpinning associations identified via genome-wide association studies of conventional phenotypes.

Psychological and behavioral traits are under a considerable degree of genetic influence, with typical heritability statistics in the 0.40–0.60 range, but specific genetic variants associated with these traits have proved elusive. Since the first molecular genetic studies in the 1990s, there have been numerous reports of genetic association that have failed to consistently replicate. Early candidate gene studies selected variants for investigation on the basis of the known or presumed neurobiology of the trait of interest. However, it soon became clear that these studies were not delivering reliable associations—initial findings were typically followed by a collection of replications, partial replications, and nonreplications, so that over time the strength of evidence for any given association tended to decline.

In stark contrast, genome-wide association studies (GWAS) have been extremely successful in identifying genetic variants associated with a range of complex phenotypes. In less than a decade, several loci associated with various complex phenotypes have been identified, through large consortium-based efforts (Tobacco & Genetics Consortium, 2010; Wellcome Trust Case Control Consortium et al.,2010). This success arose indirectly out of the stringent statistical standards imposed by the multiple testing burden inherent in GWAS—given the very large number of variants tested, a significance threshold of *p* < 5 × 10^−8^ was required. This necessitated very large sample sizes, often through international collaboration in the form of large, multistudy consortia. Replication of initial findings also became commonplace. Nevertheless, despite this success, variants identified to date via GWAS explain less than half the heritability of complex phenotypes estimated by twin and family studies. This has been described as the “missing heritability” problem (Manolio et al.,2009).

Throughout both the candidate gene and GWAS eras, a persistent topic of debate has been the extent to which the search for genetic loci might be facilitated by the use of intermediate phenotypes (sometimes described as endophenotypes, although this term has a more specific definition). These are phenotypes positioned somewhere between genetic variation and the downstream behavioral or psychological trait of interest. The implicit assumption of this approach is that, by focusing on phenotypes that are biologically closer to the genetic influence, genetic effects will be larger than for distal (i.e., behavioral) phenotypes. This implies that the genetic architecture (i.e., the number of loci, their effect sizes, and the way they operate) of intermediate phenotypes should be different to that of behavioral phenotypes.

One of the most important observations to emerge from GWAS is that almost all complex traits have a highly polygenic component; that is, their genetic basis consists of relatively frequent (i.e., minor allele frequency > 5%) risk alleles at a very large number of loci, each making a small contribution to variation, or disease susceptibility. We have previously argued that there is no strong evidence that genetic effects associated with intermediate phenotypes are substantially larger than those associated with behavioral phenotypes (Flint & Munafò, 2007). The evidence from the studies reported by the Minnesota Center for Twin and Family Research (MCTFR) in this special issue is consistent with this conclusion—despite employing a range of mechanistic intermediate phenotypes with known relevance to several behavioral traits, few suggestive signals have been observed. The likelihood is that the sample size available was simply insufficient to enable the reliable detection of common variants. Importantly, a method that uses the genotypes from a study to estimate heritability (genome-wide complex trait analysis, or GCTA) also confirms this general conclusion—a substantial proportion of the heritability of these phenotypes can be accounted for by a very large number of common variants exerting very small effects. In other words, intermediate phenotypes cannot be assumed to guarantee large genetic effects.

This is consistent with findings from GWAS of other potential intermediate phenotypes. One example is brain structural variation—similar differences in brain structure have been found in unaffected individuals at increased genetic risk of psychiatric illness and affected individuals (Brans et al.,2008; Harms et al.,2010; Pol et al.,2012). These phenotypes have been subject to GWAS in a large-scale consortium (Enhancing Neuro Imaging Genetics through Meta-Analysis: http://enigma.ini.usc.edu). Critically, the loci identified “have comparable effect sizes to those observed in other genome-wide association studies of complex traits” (Stein et al.,2012). One marker explains just 0.58% of intracranial volume per risk allele and required 21,151 participants (combined cases and controls in discovery and replication samples) to be identified (Stein et al.,2012). Mapping of measures of cognitive performance (Need et al.,2009) similarly shows genetic effects no larger than those found for psychiatric disease.

This conclusion has important implications for future GWAS projects of behavioral phenotypes, and the utility of intermediate phenotypes for gene discovery. Given that most intermediate phenotypes are more laborious and costly to collect than traditional self-report measures, they are unlikely to be deployable on the scale required by GWAS. Despite this, the limited understanding of the origins of behavioral and psychological traits makes the acquisition of intermediate phenotypes that capture the mechanisms underlying mechanistic processes essential to the interpretation of genetic findings. In other words, we expect that the best use of intermediate phenotypes will not lie in aiding gene identification but in interpreting the results of GWAS, through targeted follow-up studies of genes identified via large-scale GWAS using traditional self-report phenotypes, perhaps focusing on extreme homozygotes in order to increase statistical power (Ware, Timpson, Smith, & Munafò, 2014). This process is conceptually no different from the acquisition of physiological information to interpret a molecular explanation of disease origin. Intermediate phenotypes therefore have an important place in genetic research, but are better suited to understanding mechanistic pathways than to gene discovery projects.

There are already examples of this approach being applied fruitfully. Variants in the *CHRNA5-A3-B4* gene cluster have been reliably associated with heaviness of smoking (Ware, van den Bree, & Munafò, 2011, 2012). This finding was striking because it implicated nicotinic acetylcholine receptors not previously thought to play a major role in tobacco dependence. However, the amount of nicotine extracted per cigarette can vary considerably across individuals (e.g., due to differences in depth of inhalation, McNeill & Munafò, 2012). Therefore, biomarker assessments of heaviness of smoking are considerably more precise than self-report measures. Consistent with this, a single nucleotide polymorphism in the *CHRNA5-A3-B4* gene cluster is associated with 1% of phenotypic variance in cigarettes per day, but 4% of variance in cotinine levels (the primary metabolite of nicotine; Keskitalo et al.,2009; Munafò et al., 2012).

Genome-wide association studies continue to grow in size, through the formation of large, international consortia (e.g., the Psychiatric Genomics Consortium: http://www.med.unc.edu/pgc; the GWAS and Sequencing Consortium of Alcohol and Nicotine use: http://gscan.sph.umich.edu; and the ENIGMA-EEG Consortium: http://enigma.ini.usc.edu/ongoing/enigma-EEG-working-group, all of which involve MCTFR participation). With increasing sample size will come an increasing number of variants identified, whose function will often be unclear. These variants will then become candidates for further interrogation, where we can be confident that there is a robust association with the downstream disease phenotype. In our opinion, it is here that intermediate phenotypes may prove valuable.
